# Brain Neural Underpinnings of Interoception and Decision-Making in Alzheimer's Disease: A Narrative Review

**DOI:** 10.3389/fnins.2022.946136

**Published:** 2022-07-11

**Authors:** Weiyi Sun, Daisuke Ueno, Jin Narumoto

**Affiliations:** Department of Psychiatry, Graduate School of Medical School, Kyoto Prefectural University of Medicine, Kyoto, Japan

**Keywords:** interoception, decision-making, Alzheimer's disease, anosognosia, somatic marker hypothesis, reinforcement learning, predictive coding

## Abstract

This study reviews recent literature on interoception directing decision-making in Alzheimer's disease (AD). According to the somatic marker hypothesis, signals from the internal body direct decision-making and involve the ventromedial prefrontal cortex (vmPFC). After reviewing relevant studies, we summarize the brain areas related to interoception and decision-making (e.g., vmPFC, hippocampus, amygdala, hypothalamus, anterior cingulate cortex, and insular cortex) and their roles in and relationships with AD pathology. Moreover, we outline the relationship among interoception, the autonomic nervous system, endocrine system, and AD pathology. We discuss that impaired interoception leads to decreased decision-making ability in people with AD from the perspective of brain neural underpinning. Additionally, we emphasize that anosognosia or reduced self-awareness and metacognition in AD are remarkably congruent with the malfunction of the autonomic nervous system regulating the interoceptive network. Furthermore, we propose that impaired interoception may contribute to a loss in the decision-making ability of patients with AD. However, there still exist empirical challenges in confirming this proposal. First, there has been no standardization for measuring or improving interoception to enhance decision-making ability in patients with AD. Future studies are required to better understand how AD pathology induces impairments in interoception and decision-making.

## Introduction

Sensation helps humans understand the entire world. Through sensation, humans can observe beautiful scenery, listen to soothing music, feel a cool breeze, taste a cup of hot coffee, etc. Sight, hearing, touch, smell, and taste are examples of exteroception sensations, which occur when people receive stimuli from the outside world (Bigley, 1990; Bechara and Damasio, 2005). Additionally, sensations can come from the body itself. For example, if a person's heartbeat becomes painfully rapid, they may become tense. Such feeling from within the body is referred to as interoception; it constitutes afferent sensory information arising from the sensation, perception, and awareness of the afferent feedback from the viscera, which maintains the homeostatic function (Craig, 2002). Recently, the concept of interoception has been expanded to include not only the perception of bodily states by afferent processing but also the process of control in which the bodily state is altered in response to environmental demands while maintaining homeostasis through efferent processing, through the activity of the neuroendocrine and immune systems (Chen et al., 2021). Moreover, interoception aids in the continual anticipation of metabolic needs so as to satisfy them before they arise (referred to as “allostasis”) (Sterling, 2012) as it plays a role in choosing the appropriate action (i.e., decision-making) through monitoring and regulating one's bodily state (Quigley et al., 2021).

Neural pathways for signals from the internal body to the brain can be broadly divided into the spinal cord–brainstem–thalamus pathway and the vagus nerve pathway (Chen et al., 2021). The chemoreceptors, humoral receptors, mechanoreceptors, osmoreceptors, among others in various parts of the body provide information about the state of organs, blood vessels, muscles, and skin to these two neural pathways (Berntson and Khalsa, 2021). Interoception in the spinal cord–brainstem–thalamus pathway first travels to the dorsal column of the spinal cord through the dorsal root ganglion. Through spinal afferents, sometimes referred to as “sympathetic afferents,” interoception then moves to the rostral ventrolateral medulla and the paraventricular nucleus in the brainstem and thalamus. The neural pathway then extends from the thalamus to the posterior insula, anterior cingulate cortex (ACC), amygdala, and striatum. Conversely, interoception in the vagus nerve pathway first travels to the nucleus tractus solitarii in the brainstem through the nodose and jugular ganglion, sometimes referred to as “parasympathetic afferents.” The signal of this pathway moves to the insula, ACC, amygdala, and striatum through the thalamus as in the spinal cord–brainstem–thalamus pathway. There is also a pathway that follows the spinal cord–brainstem–thalamus and the vagus nerve pathways (i.e., from the body to the brain, afferent/ascending neural pathway) in reverse, from the brain to the body (efferent/descending neural pathway) (Chen et al., 2021).

The brain perceives and integrates signals from the internal body, providing a map of the internal landscape to induce inner sensation. Under certain conditions, such cross-talk induces impulsivity, sensory drive, and emotional experience, emphasizing the importance of internal sensation in maintaining homeostasis, bodily regulation, and survival (Khalsa and Lapidus, 2016). As stated in a previous study, an important aspect of internal sensation is the ability to maintain homeostasis and optimize the enjoyment experience by directing future behavior decisions (Furman et al., 2013). The insular and cingulate cortices control homeostasis-related brainstem regions such as the periaqueductal gray matter and are involved in the regulation of physical states (Craig, 2002). The posterior insular cortex sends integrated interoceptive signals to the anterior insular cortex, which in turn influences the conscious experience of bodily sensations, including the timing of the heartbeat.

Studies on interoception have also examined subjective awareness of heartbeat sensations and have found interoceptive awareness is mediated not only by visceral afferents transmitting to the insula and ACC but also by skin afferents transmitting to the somatosensory cortex (Khalsa et al., 2009b). Interoception as the sensation of the timing of the heartbeat (called “interoceptive accuracy”) is usually measured using electrocardiography (ECG) or electroencephalography, and thus, most information is from cardiac interoceptive studies. To measure interoceptive accuracy, three methods are generally used: the heartbeat counting task (Schandry, 1981), heartbeat detection task (Brener and Jones, 1974), and heartbeat-evoked potential (HEP). In the heartbeat counting task, participants are asked to count their number of heartbeats during specific intervals, while in the heartbeat detection task, they are asked to distinguish between signals (auditory or tactile stimuli) that are either synchronized or unsynchronized with the R wave on an ECG. HEP is a cortical response in the brain to heartbeats that occurs 200–600 ms after the R wave of the ECG waveform (Schandry et al., 1986). Many studies have shown that HEP correlates strongly with interoceptive accuracy (Schandry et al., 1986; Fukushima et al., 2011).

Numerous studies have shown age-related decreases in the brain regions related to interoception, namely, the primary somatosensory cortex (Good et al., 2001; Sowell et al., 2003; Salat et al., 2004) and insular cortex (Good et al., 2001; Resnick et al., 2003). However, a few studies have reported that interoceptive accuracy is negatively correlated with age (Khalsa et al., 2009a; Murphy et al., 2018). Ueno et al. (2020) reported that interoceptive accuracy involved positively functional connectivity in the insular, anterior cingulate, and orbital frontal cortices seeded by the rostral prefrontal cortex in older adults.

A large number of psychiatric disorders [e.g., anxiety (Paulus and Stein, 2010), panic disorder (Van Diest, 2019), obsessive-compulsive disorder (Yoris et al., 2017), and depersonalization (Sedeño et al., 2014)], somatic symptom disorders (Witthöft et al., 2020), neuro-developmental disorders [e.g., attention-deficit hyperactivity disorder (Kutscheidt et al., 2019)], autism spectrum disorders (DuBois et al., 2016), and eating disorders [e.g., anorexia nervosa (Jacquemot and Park, 2020) and bulimia nervosa (Klabunde et al., 2017)], as well as depression (Paulus and Stein, 2010), posttraumatic stress disorder (Nicholson et al., 2016), and substance use disorders (Paulus et al., 2013), have all been linked to interoceptive dysfunction. Although few in number, several studies have confirmed the association between interoceptive dysfunction and neurodegenerative diseases, including Alzheimer's disease (AD) (García-Cordero et al., 2016; Salamone et al., 2021), behavioral variant frontotemporal dementia (García-Cordero et al., 2016; Salvato et al., 2018; Salamone et al., 2021), and Parkinson's disease (Salamone et al., 2021). In addition, the topic of interoception has received much scholarly attention in recent years. This is partly because interoception has an important influence on cognition and partly because it can guide decision-making and alter memory and emotional processes (Critchley and Harrison, 2013).

Regarding AD, there are two types of neuropathological changes that can be used to predict disease progression: One is “positive” lesions, such as amyloid plaques, neurofibrillary tangles, dystrophic neurites, neuropil threads, and other deposits accumulated in the brain of patients with AD, and the other is “negative” lesions, such as significant atrophy caused by neuronal loss and synaptic loss in the brains of patients with AD (Serrano-Pozo et al., 2011). Pathological analysis of AD shows that the limbic system, particularly the hippocampus and amygdala, is severely and often affected (Hopper and Vogel, 1976). Retrograde amnesia caused by damage to the hippocampus is the best-known symptom of AD.

The decision-making abilities of patients with AD deteriorate as their cognition decreases, and interoceptive functions may play an important role in this progression. Currently, few studies have focused on interoception in patients with AD, including the occurrence, development, and significance of interoceptive impairment in these patients. Thus, in this narrative review, we investigate the impact of interoception on the decision-making ability of patients with AD. To the best of our knowledge, this is the first study to examine findings on the causes of reduced decision-making ability in patients with AD from the perspective of interoception.

## Purpose of Interoception in Directing Decision-Making

### Somatic Marker Hypothesis: Theories

Damasio's somatic marker hypothesis (SMH) was the first to suggest the notion of interoception directing decision-making. This hypothesis states that the brain may respond to specific content that is associated with certain objects and events, which can be actual or recalled. The brain's response to content can result in a number of changes in the body or brain state. Emotion is seen as the sum total of changes (Damasio, 2008). According to Damasio (1996), when stimulation occurs, including muscle tension, heartbeat, endocrine activities, and facial expressions, the brain and body's overall emotional changes are transmitted to the brain, where they cause the release of neurotransmitters, changes in the state of the somatosensory cortex such as the insula, and transmission of signals from the body to the somatosensory cortex.

This hypothesis proposes two distinct pathways for reactivating the somatic marker response (Bechara and Damasio, 2005): The first pathway is the “body loop,” which involves physical changes transmitted to the brain that can directly induce emotions, and the other pathway is the “as-if body loop,” which allows the cognitive representation of emotions to be activated in the brain without the need for direct sensory stimulation. When a scene or memory is linked to a previous experience, the “as-if body loop” is activated, and the brain combines the information into the corresponding internal somatic state, causing internal changes in the body and eliciting a bodily response. As a result, when faced with a difficult decision, the emotions caused by the somatic state tend to favor one's return to a previous happy experience or the avoidance of a painful experience similar to the past.

### SMH Model

Primary inducers are innate or learned sensory stimuli that can be pleasurable or averse, automatically and compulsively triggering a physical response (Bechara and Damasio, 2005). Secondary inducers are entities generated by recollections of personal or hypothetical emotional events associated with primary inducers.

Bechara and Damasio (2005) hypothesized that in the “body loop” pathway, primary inducers stimulate the somatic pattern mainly through the amygdala and a small part of the ventromedial prefrontal cortex (vmPFC). The amygdala can combine the features of primary inducers with the somatic state associated with the inducer, which can be processed subliminally (e.g., *via* the thalamus) or overtly (e.g., *via* early sensory and high-order association cortices). At the same time, the activity of the amygdala and the vmPFC can cause effector activity, which can stimulate the body and generate a specific somatic state. In the end, the specific somatic state is transferred up the insula and nuclear neurotransmitters, causing the central nervous system to release transmitters.

In the “as-if body loop” pathway, the vmPFC is considered a key region for secondary inducers to trigger somatic states (Bechara and Damasio, 2005). After the primary trigger activates the body model, an inner model is usually formed in the brain. When the secondary inducers, namely, the memory of the primary inducers, appear, the vmPFC rapidly combines the information of the secondary inducers into the relevant inner model without passing through the body to the brain.

### SMH Verification

The first examples of impaired decision-making in the studies selected for this review were in patients with damage to the vmPFC (Bechara et al., 1994). Such people retain average intelligence and memory but cannot make proper decisions in daily life. Patients with vmPFC impairment make decisions that do not appear to be in their long-term interest, exhibiting decision-making patterns described as future myopia due to a lack of trade-offs between short- and long-term consequences (Bechara et al., 2000; Bechara, 2001).

To successfully determine the decision-making abilities of such patients, Damasio et al. designed the Iowa Gambling Task (IGT) (Bechara et al., 1994). In this task, individuals are given $2,000 to begin with and asked to choose cards from one of four decks to maximize profit over the course of 100 trials. Decks A and B produce an average profit of $100 per draw, whereas decks C and D produce an average profit of $50 per draw. Individuals incur a net loss of $250 after 10 selections from decks A and B, whereas they incur a net gain of $250 after 10 selections from decks C and D. In the long run, decks A and B are termed “disadvantageous,” and selection from these decks is deemed risky, while decks C and D are termed “advantageous,” with selection from these decks providing benefits (Bechara et al., 1994). To make the best gambling decisions, a person must renounce significant occasional wins to accumulate modest gains that are more advantageous in the long term. Patients with vmPFC impairment frequently choose solutions that yield huge short-term rewards but eventually result in long-term losses. Healthy participants rapidly learn that picking the alternatives with fewer short-term profits yields the best long-term results.

A number of studies employing skin conductance response (SCR) as a measure of affective state have shown a link between poor decision-making in the gambling task and affective, emotional reactions (Bechara et al., 1996, 1997, 1999). Both the vmPFC impairment group and normal control group showed an SCR at the start of the gambling task, when participants learned the option-related outcomes. However, in the later session, the normal control group produced a kind of expected SCR that was present when they were able to understand the instructions attached to the options; contrarily, the patients with vmPFC impairment did not develop a prospective SCR. Thus, the SCR can be used to predict choice and bias of future decisions, which provides evidence for the SMH.

However, the IGT confirmation of the SMH is not supported by all research studies. Maia and McClelland (2004) believe that participants' favorable decisions seem to stem from conscious knowledge of the advantageous strategy. The poor performance of patients with vmPFC in the IGT may be due to the difficulty of adjusting to the reversals in contingencies. Furthermore, Dunn et al. (2006) found that the task design is not rigorous because in the IGT, the influence of working memory or cognition cannot be excluded. There is also no theoretical support for the idea that learning takes place through anticipatory marker signals produced by the body. In addition, the task obstacles can be explained by more credible psychological mechanisms (such as reverse learning and working memory defects). Nevertheless, the neural substrate of the SMH proposed by Damasio (1994) has now been fully confirmed by research results, and the IGT is also widely used in fields related to emotion and decision-making (Chiu et al., 2018).

## Interoception and Decision-Making in AD

Although the theory that interoception is associated with decision-making is widely supported, as seen in the SMH, it is unclear how interoception is linked to decision-making. Recently, Keramati and Gutkin (2014) described the association between interoception and decision-making based on interoceptive prediction (Seth et al., 2012; Barrett and Simmons, 2015; Seth and Friston, 2016). Interoceptive prediction posits that predictive processing, as proposed by Friston (2010), also occurs in interoception. To maintain homeostasis, the brain constructs an internal model to achieve the desired body state. This model predicts optimal values according to environmental demands, and the difference between the actual body state and the prediction is detected as a prediction error. The individual tries to maintain homeostasis by efficiently minimizing this prediction error. According to the theory of interoceptive prediction, interoception functions as a signal that tells us whether the prediction error is within or outside the acceptable range, which directs decision-making to maintain the prediction error within the acceptable range.

Keramati and Gutkin (2014) devised a homeostatic reinforcement learning model that hypothesizes that animals are capable of predicting the impact of behavior-related outcomes on their internal state; they find a behavior rewarding if they believe that the predicted impact of its outcome will reduce prediction error. Their model shows that animals stabilize their internal state around the ideal value by simply learning to perform behaviors that lead to rewarding outcomes. Indeed, HEP amplitude in the vmPFC is strongly correlated with preference value decision-making (Azzalini et al., 2021), and cardiac afferent signals, only in the systole phase but not in the diastole phase, enhance asymmetric value updating based on reinforcement learning (Kimura et al., 2022).

We discussed the decrease in the decision-making ability of patients with AD in our previous article (Sun et al., 2021). We found their decision-making ability was influenced by both internal and external factors. Internal factors included brain changes, physiological changes, cognitive impairment, and emotional changes, while external factors included information complexity, cultural values, and the decision-making situation. García-Cordero et al. (2016) reported that patients with AD showed lower interoceptive accuracy and learning alongside higher interoceptive awareness than healthy participants. Moreover, they reported that interoceptive deficits were associated with atrophies of not only gray matter volume in insular and cingulate cortices but also the hippocampus and temporal regions in patients with AD. Therefore, interoceptive deficits in patients with AD seem to rely more critically on general memory and learning skills. Additionally, Salamone et al. (2021) reported that patients with AD maintain interoception but have lower negative and neutral face recognition than a healthy control group and suggested HEP modulations occur during emotional face recognition. The same study also found HEP variance in patients with AD was large. Thus, emotional processing is deficient in patients with AD (Christidi et al., 2018), so future research should further investigate interoception in these patients.

Based on these data, we hypothesize that the prediction error in interoception in AD may be due to dysfunction of interoceptive afferent signals, which may decrease decision-making ability, such as preference choice or value updating. As mentioned, the SMH proposes that internal sensation is related to brain regions, namely, the amygdala, vmPFC, thalamus, and insula. As a result, disturbances in any of these areas, which are associated with internal sensation, general memory, and learning skills, can lead to an impairment of interoception in patients with AD. However, as far as we know, the role of interoception in decision-making in patients with AD has not been sufficiently examined.

### Role of the vmPFC

The vmPFC is a part of the prefrontal cortex in the human brain, and it is involved in regulating and controlling emotions (Damasio et al., 1990; Damasio, 2003) and directing decision-making (Denburg et al., 2007), as well as controlling human's moral ideas (Hu and Jiang, 2014).

McCormick et al. (2020) proposed that the vmPFC activates earlier than the hippocampus during the initiation of autobiographical memory (AM) recall, except during retrieval of the most recent AMs. They also found that the vmPFC drives hippocampal activity at recall commencement, as well as the unfolding of AMs over the following seconds, independent of AM age (McCormick et al., 2020). Therefore, the ability of the vmPFC to drive the hippocampus may be consistent with the suggestion of the SMH that the vmPFC is an important brain region capable of triggering the somatic state from secondary inducers (Bechara and Damasio, 2005). When the vmPFC receives emotional stimuli from secondary inducers, the somatic state is activated, and the related information is transmitted to the amygdala or higher brain structures, such as the brainstem nuclei and hypothalamus (Damasio et al., 1990; Damasio, 1996). Despite the amygdala activity being increased, patients with vmPFC injury experience reduced cardiac deceleration when viewing aversive images (Motzkin et al., 2015). As a result, there is evidence that the vmPFC controls autonomic responses to emotion.

The frontal aging hypothesis is one of the most important theories to explain how the brain changes, leading to AD. This hypothesis states that the frontal brain regions have an accelerated deterioration with aging compared to other brain regions, resulting in a decay of frontal brain functions (West, 1996). Degenerative changes in specific areas of the brain, including the temporal and parietal lobes and restricted regions within the frontal cortex and cingulate gyrus, may underlie the specific aspects of the dementia associated with AD (Wenk, 2003). In Stoeckel et al. (2013), atrophy of the medial prefrontal cortex and the corresponding decline in attention were related to decreased financial capacity. This may serve as evidence indicating that interoception plays an important role in directing decision-making in people with AD.

### Role of the Limbic System

The limbic system is a group of brain structures, including the limbic cortex (cingulate gyrus and parahippocampal gyrus), hippocampal formation (the dentate gyrus, hippocampus, and subicular complex), amygdala, septal area, and hypothalamus, which are essential brain structures for forming a network to control emotion (Rajmohan and Mohandas, 2007).

The limbic system is thought to be involved in processes that regulate emotion and motivation. Papez (1937) proposed an emotional circuit controlled by the cerebral cortex by injecting a rabies vaccine into the hippocampus of cats and observing its progress in the brain. According to Papez (1937) theory of the central emotive process of the cortical origin, emotions are established during hippocampal formation. Subsequently, they are transmitted to the mammillary bodies through the anterior thalamic nucleus to the cingulate gyrus and back to the hippocampal formation through the entorhinal cortex. He revealed that emotion is a physiological process whose entire emotional circuitry is, in part, mediated by the hypothalamus, which integrates sensory information from the gut and connects with the autonomic nervous system and endocrine system by controlling the hypophysis (Rinaman, 2007). Therefore, the hypothalamus is the high-level center for regulating visceral activity and the endocrine system and is an indispensable structure for the formation of interoception.

The amygdala seems to be a major brain structure in processing emotion. It comprises multiple subnuclei that control different functions, among which the basolateral amygdala (BLA) and central nuclei (CeA) are considered the two most important subnuclei involved in emotional processing. The BLA can receive sensory information and use learned information to control the CeA. As a result of receiving information from the BLA or parallel input from the cortical and subcortical structures, the CeA extensively transmission to the hypothalamus, midbrain reticular structure, and brain stem and can regulate and control the body's behavior, autonomic response, and neuroendocrine system (Cardinal et al., 2002). As mentioned in the SMH, the amygdala is seen as the region where primary inducers trigger the somatic state, as well as the one that relates emotions to emotional events (Bechara and Damasio, 2005).

Recent functional neuroimaging studies have shown that both the insula and anterior cingulate gyrus are activated in response to interoceptive stimuli, such as the heartbeat and gastrointestinal sensations (Critchley et al., 2004; Van Oudenhove et al., 2004; Pollatos et al., 2007). The anterior cingulate gyrus is connected to the brainstem, amygdala, hypothalamus, and orbitofrontal cortex (Yasui et al., 1991; Floyd et al., 2000), playing an important role in the production of motivation and emotion (Craig, 2002). Specifically, the feeling is formed by the insula, motivation is formed by the anterior cingulate gyrus, and emotion is formed by the two (Craig, 2002). This could be because the primary function of the anterior cingulate gyrus is to generate visceral motor predictive signals of the body's internal milieu, which then regulate and guide the subcortical nuclei (e.g., hypothalamus and brainstem) and modulate the autonomic nervous, endocrine, and immune systems (Smith et al., 2019). Predictive signals are generated in the posterior or mid-insula, being constrained and updated by comparing them with incoming signals that convey the true state of the body's systems (Smith et al., 2019). Nevertheless, damage to the temporal lobe and limbic system can occur, even in the early stage of AD and in mild cognitive impairment patients. As a result, in patients with AD, damage from the limbic system may lead to decreased interoception and thus affect decision-making ability.

### Role of the Insula

The insula is a brain structure and a part of the cortex. It is folded deep within each hemisphere's lateral sulcus, hidden beneath the opercula, or “lids,” which are made up of parts of the frontal, parietal, and temporal lobes (Gogolla, 2017).

The insula has a variety of functions. It can receive sensory information and process it as an integral brain hub, transmitting the collected information to other brain areas. Because of having connections to other brain structures such as the amygdala and hypothalamus (Ibrahim et al., 2019), the insula helps form bodily feelings and control the autonomic nervous system to regulate the heart rate and blood pressure (Shoemaker and Goswami, 2015). Craig (2002) was the first to propose that the insula is a critical brain region that underpins interoception; building on this idea, Farb et al. (2012) proposed that the posterior insula is the region for receiving interoception signals from the thalamus. In 2005, Bechara and Damasio reaffirmed the role of the insula in interoception and conducting decision-making. They suggested that the insula can be reactivated, along with the vmPFC and amygdala, as part of somatic-state patterns when subjects are evaluating familiar stimuli (Bechara and Damasio, 2005). This may be because the anterior insula integrates interoceptive information from the posterior insula with exteroceptive information, thus connecting the internal body state to the resultant effects of external stimulation (Farb et al., 2012). Benarroch (2020) reported that the posterior insula cortex projects to the anterior insula, which integrates interoceptive signals and is involved in the conscious experience of bodily sensation. In the interoceptive predictive coding model, the anterior insula is thought to be the most important target because it is involved in both interoception and emotion generation (Seth et al., 2012). This interactive process in the insula plays a major role in interoception by issuing prediction signals on the expected state of the body based on previous experience.

Usually, the first part of the brain affected by pathological changes in patients with AD is the entorhinal cortex (Braak and Braak, 1995; Khan et al., 2014). As the disease progresses, changes occur in the limbic system near the entorhinal cortex, including the hippocampus and amygdala, and then pathological changes develop in the entire neocortex (Braak and Braak, 1995). Similarly, the pathological changes in the insular cortex are also related to the disease progression in patients with AD. According to Bonthius et al. (2005), the insular cortex is affected by pathological changes in patients with AD, and a series of manifestations appear, including autonomic dysfunction, visceral sensory changes, destruction of self-happiness, and impairment of decision-making. Therefore, sensation occurs in the human body *via* the insula, and because of how the disease progresses in patients with AD, decision-making ability might become impaired as the insula function deteriorates.

### Role of the Autonomic Nervous System and Endocrine System

Interoception, or the perception of the body's physiological state, aids homeostatic control and allostatic adaptation by ensuring the organism's stability (Berntson et al., 1993) and directing behavior through sensations like hunger, thirst, and dyspnea. This dynamic balance of interoception depends on the brain. The brain can not only perceive interoceptive information from the body through an ascending pathway but can also regulate the body to maintain internal balance from a descending pathway. Moreover, the peripheral nervous and central nervous systems, as well as components of the vascular, endocrine, and immunological systems, are all engaged in processing signals about the internal environment (Chen et al., 2021).

Through the autonomic nervous system, interoception signals from the peripheral nervous system are transmitted to the central nervous system in two different pathways. These two pathways use two different types of peripheral sensory ganglia to transfer signals. Nodose or jugular ganglia, which are found along the cranial/vagal pathways, frequently transmit to the nucleus tractus solitarii of the brainstem, whereas dorsal root ganglia, which are placed along the spinal nerve route, provide signals to the brain through the spinal cord (Craig, 2002). This visceral pathway through the cranial nerves, including the vagus nerve, is called the parasympathetic pathway, and the pathway through the spinal cord is called the sympathetic pathway (Mei, 1983).

In addition to neural pathways, the body can also modulate interoceptive signals through non-neural pathways (e.g., humoral) (Carvalho and Damasio, 2021; Chen et al., 2021). In such pathways, regulatory signals, which are sent to the peripheral organ *via* the vascular or lymphatic systems, interact directly with the responding non-neural cells. In the interoceptive apparatus, non-synaptic neuronal transmission is common. Unlike synaptic neuron transmission, it is dependent on neurotransmitters such as monoamines (dopamine, noradrenaline, and serotonin), acetylcholine, and neuropeptides. Interoception is “fluid” and “continuous,” and it is because of the transmission of non-synaptic neurons that the central nervous system of interoception is in close and continuous contact with the body itself, providing a basis for the formation of sensation (Carvalho and Damasio, 2021).

In the field of physiology, the theory that neurotransmitters such as dopamine (Mohr et al., 2010; Eppinger et al., 2011; Samanez-Larkin and Knutson, 2015), serotonin (Mohr et al., 2010; Eppinger et al., 2011), norepinephrine (Mohr et al., 2010; Samanez-Larkin and Knutson, 2015), and glutamine (Samanez-Larkin and Knutson, 2015) influence decision-making has also been proposed, and supporting evidence has been presented. To explain the participation of emotional and motivational conduct in decision-making, Samanez-Larkin and Knutson (2015) presented the affect–integration–motivation framework, which includes neurons such as dopaminergic, noradrenergic, and glutamatergic neurons. According to some researchers, the occurrence of AD may be caused by synaptic plasticity, which is mediated by glutamate and its receptors (Wang and Reddy, 2017). Similarly, the progression of AD may be influenced by changes in dopamine system neurosecretion (Pan et al., 2019), as well as the degeneration of the locus coeruleus noradrenergic neurons, which have been linked with impairments in global cognition, episodic memory, working memory, and visuospatial ability (James et al., 2021). In this case, the change in decision-making progress in AD may be due to a decline in neurological function, as well as physiological alterations.

## Discussion

**Figure 1:**
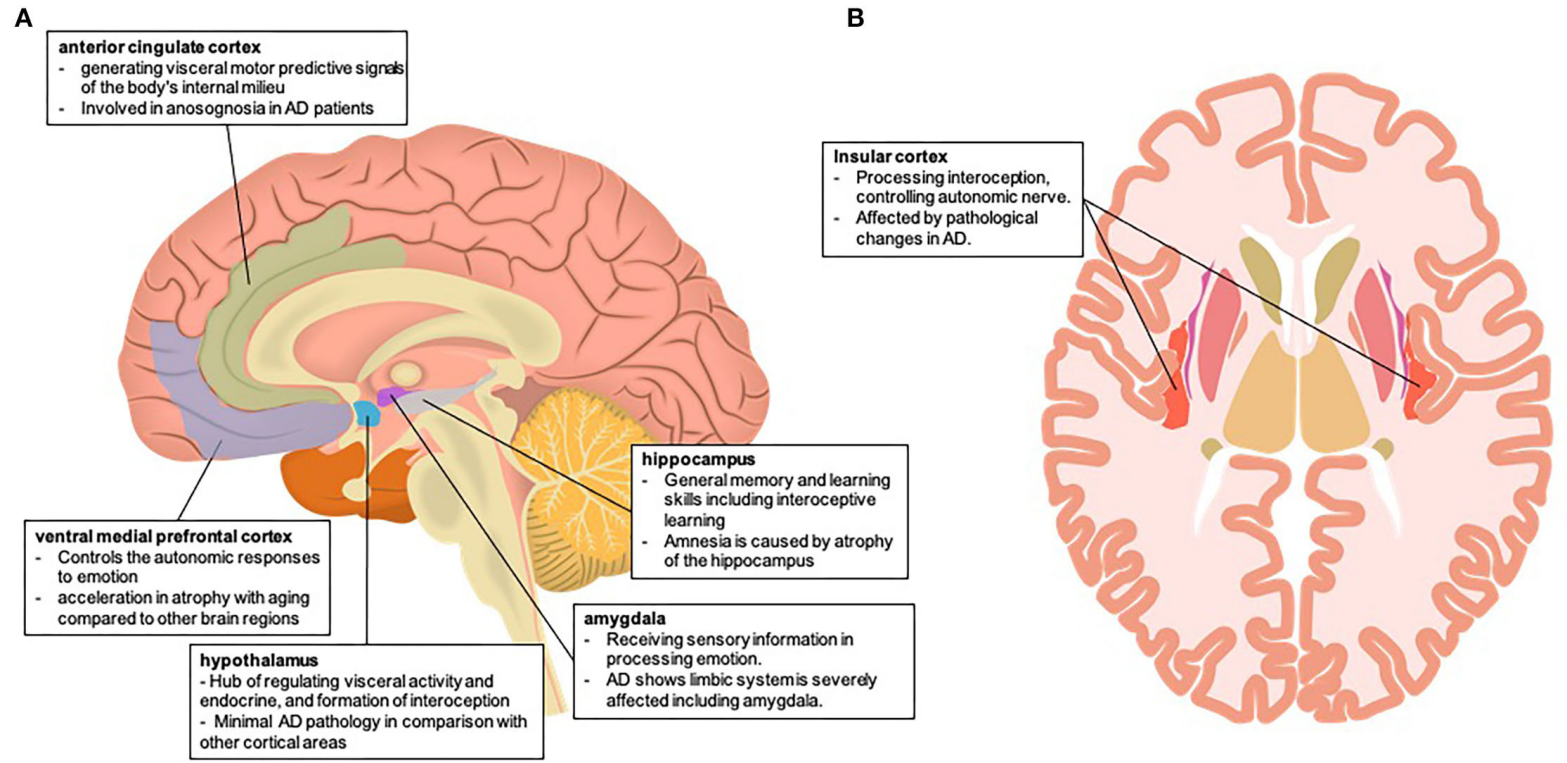
Brain neural mechanism of impaired interoception in Alzheimer's disease **(A)** involving the brain area of interoception and the autonomic nervous system in the mid-sagittal section **(B)** involving the brain area of interoception and the autonomic nervous system in the horizontal section. Both brain illustrations were used and modified from the following site: **(A)** Database Center for Life Science (DBCLS), mid-sagittal plane of the brain, February 1, 2021, doi:10.7875/togopic.2021.023 **(B)** Database Center for Life Science (DBCLS), Horizontal plane of the brain, February 1, 2021, doi:10.7875/togopic.2021.026.

The present article discusses the possibility of impaired interoception leading to decreased decision-making ability in people with AD from the perspective of brain neural mechanisms (Figure 1). The establishment of an interoceptive network in the human body depends on both neural and non-neural systems. The composition of the neural system depends on the regulation of the hypothalamus and autonomic nervous system by brain structures, including the vmPFC, amygdala, hippocampus, thalamus, and insula, and the non-nervous system, including the immune and endocrine systems. In patients with AD, the brain structures and neurotransmitters involved in the interoceptive networks are often affected by pathology. Therefore, the consistency of this interoceptive network with the pathological lesion site of AD may provide the possibility that interoception dysfunction leads to a decreased decision-making ability in patients with AD.

Neural monitoring of internal body signals may play a role in self-awareness. Babo-Rebelo et al. (2016) highlighted that there are two distinct self-dimensions coded by the default brain network in neural responses to heartbeats, that is, “I” and “Me,” representing the cognitive self and bodily self, respectively. The unifying mechanism behind the cognitive and bodily selves is neural monitoring from the internal organs, rather than representational convergence on a given brain area. Therefore, self-awareness is largely dependent on interoception.

A study of human autonomic psychophysiology suggests that cognition, emotion, and internal states of bodily arousal are integrated and undergo bidirectional coupling. As such, the brain centers that support perception, memory, thoughts, and feelings are closely related to changes in the inner physiological bodily states that represent autonomic regulation. Prediction processing determines the subjective sensory state; in other words, the predictive model predicts the interoceptive response to external stimuli and/or internal physiological signals (i.e., emotions) (Seth et al., 2012). Such changes are essential components of emerging sensations that are thought to be the basis for emotion and motivation, as well as a coherent sense of self. Self-awareness is related to metacognition—the need to appraise thoughts themselves (e.g., knowing whether your perceptions, thoughts, and actions can be trusted) as cognitive processes increase in complexity. The close relationship between metacognition and self-awareness connects individual feelings and motivation to the brain's control (above the brainstem) over much of the autonomic function, beyond organ-specific homeostatic reflexes, to make decisions about what the individual and body as a whole should do (Quadt et al., 2022).

People with AD have a consciousness deficit, which causes them to underestimate the complexity of cognitive tasks and overestimate their ability to remember, despite the fact that assessments of their family members' cognitive abilities remain consistent with those of normal people (McGlynn and Kaszniak, 1991). In fact, participants with AD report overestimating their ability to be aware of their interoception, despite a lower accuracy of their actual heartbeat than that of a healthy control group (García-Cordero et al., 2016). This inability to recognize one's own neurological and psychiatric impairment (i.e., motor, sensory, cognitive, or behavioral) is called anosognosia. In the context of AD, anosognosia is also known as impaired self-awareness and includes milder forms of anosognosia (Prigatano, 2009). A review summarizes eight brain regions that may be involved in anosognosia in patients with AD, including the inferior frontal gyrus, anterior cingulate gyrus, medial temporal lobe, superior frontal gyrus, medial frontal gyrus, orbitofrontal cortex, insula, and posterior cingulate cortex (Hallam et al., 2020). Zamboni and Wilcock (2011) located the processing of self-awareness in the brain's orbitofrontal cortex, insula, and medial temporal lobe and believed that these structures simultaneously formed a part of the default mode network. The default mode network is a large-scale network, made up of interconnected brain areas, that is thought to be linked to self-related cognition tasks such as the ability to imagine future occurrences (Zamboni and Wilcock, 2011; Weiler et al., 2016). Moreover, the salience network, which involves the anterior insular cortex and ACC, is correlated with interoception (Kleckner et al., 2017), even in healthy older adults (Ueno et al., 2020). AD pathology has shown weakened connectivity within the default mode network and strengthened connectivity within the salience network, and functional compensation is a more complex process, indicating that there may be multiple ways participating in this process and not just compensation by large-scale networks (Fu et al., 2021). Thus, anosognosia, or reduced self-awareness and metacognition, in AD is remarkably congruent with a malfunction of the autonomic neural mechanism regulating the interoceptive network. This also raises the idea that impaired interoception may contribute to a loss of decision-making ability for people with AD.

## Conclusion

**Figure 2:**
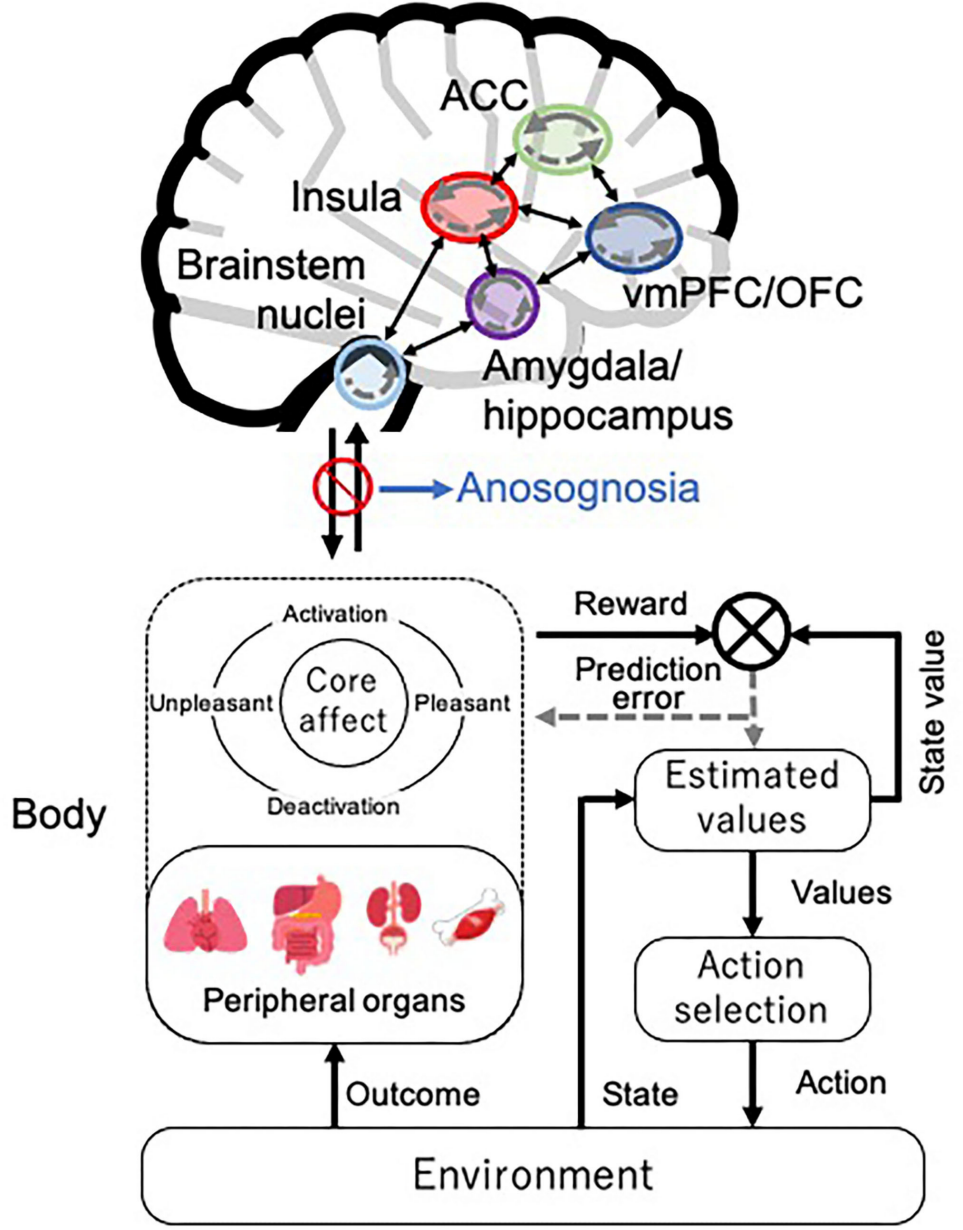
Decision-making model based on interoceptive reinforcement learning. We present the conceptual decision-making model of reinforcement learning in interoceptive prediction. The solid arrows represent the prediction signal, and the dotted arrows represent the prediction error signal within each brain region. ACC, anterior cingulate cortex; vmPFC, ventromedial prefrontal cortex; OFC, orbitofrontal cortex. The brain illustration was used and modified from the following site: Jmarchn, CC BY-SA 3.0, Human brain icon, August 7, 2020, https://commons.wikimedia.org/wiki/File:Human_brain_icon.svg.

To the best of our knowledge, this is the first review that attempts to analyze findings of previous studies on the causes of reduced decision-making ability in patients with AD from the perspective of interoception. While our previous study investigated the reasons for reduced decision-making ability in patients with AD (Sun et al., 2021), the role of interoception was not taken into account. By analyzing the theoretical background and brain neural underpinnings involved in interoception and decision-making, we found that these brain areas are often simultaneously affected by AD pathology. Decision-making is considered a reinforcement learning model for maintaining homeostasis (Keramati and Gutkin, 2014): the body constitutes the peripheral organs and emotion (core affect is assumed here) in the SMH (Bechara and Damasio, 2005) and is thought to be part of the process for optimizing prediction error through predictive processing of interoception (Figure 2). According to this conceptual decision-making model based on interoceptive reinforcement learning, the reduced decision-making ability in patients with AD would lead to impairments in optimizing their prediction error of interoceptive accuracy and updating their estimated values because of dysfunction in the vmPFC, insula, ACC, and amygdala. Moreover, the “as-if body loop” (Damasio, 1999) corresponds to a kind of internal model in predictive processing. Damasio (1999) argues that it is possible to predict the perception of bodily reactions through the “as-if body loop” and make decisions based on these predictions. Assuming that the “as-if body loop” state is anosognosia in patients with AD, these patients tend to process in a way that is heavily weighted toward the internal model. This impairs interoceptive accuracy and value updating and may lead patients with AD to frequent decision-making failures.

This review has not covered the dimensions from neuropsychology and functional magnetic resonance imaging studies, and there are still challenges in confirming the hypothesis presented herein. First, there is no standard for measuring interoception in patients with AD. Given their reduced cognitive function, some of the explanations for measurements of cognitive function are incomprehensible for them. Therefore, one possibility for measuring interoception in patients with AD is HEP. Recently, some studies have examined HEP in patients who have dementia, including AD (García-Cordero et al., 2016; Salamone et al., 2021); thus, this method may be useful to measure interoceptive accuracy in patients with AD. However, due to the lack of an effective measurement method for HEP and the inability to remove the influence of cardiac dynamics, measuring interoception by using HEP is still in its early stages (Coll et al., 2021). Second, if interoception can be measured, how can we improve interoception to improve decision-making ability? To improve interoception, Weng et al. (2021) posit neuromodulation *via* stimulation of the vagus nerve and brainstem nuclei focusing on the nucleus tractus solitarius, as well as mindfulness approaches for regulating the autonomic nervous system and slow breathing for regulating the parasympathetic nervous system. Although these approaches contribute to improving interoceptive accuracy and awareness, it is not clear this effect would occur in patients with AD. Despite many outstanding challenges, the present review still provides a potential method for assessing the decision-making ability of patients with AD from the perspective of the autonomic nervous system and interoception. As maintaining the decision-making ability of patients with AD may be beneficial both economically and medically, future empirical research is required to better understand how AD pathology induces impairments in interoception and decision-making.

## Author Contributions

WS, DU, and JN designed the study, searched and reviewed previous literature, wrote the first draft, and reviewed and critiqued the manuscript. All authors read and approved the final manuscript.

## Funding

This study was based on work supported by JSPS KAKENHI (Grant Number: 19K14454).

## Conflict of Interest

The authors declare that the research was conducted in the absence of any commercial or financial relationships that could be construed as a potential conflict of interest.

## Publisher's Note

All claims expressed in this article are solely those of the authors and do not necessarily represent those of their affiliated organizations, or those of the publisher, the editors and the reviewers. Any product that may be evaluated in this article, or claim that may be made by its manufacturer, is not guaranteed or endorsed by the publisher.
